# Detection of activating and acquired resistant mutation in plasma from EGFR-mutated NSCLC patients by peptide nucleic acid (PNA) clamping-assisted fluorescence melting curve analysis

**DOI:** 10.18632/oncotarget.17786

**Published:** 2017-05-10

**Authors:** Chang Gon Kim, Hyo Sup Shim, Min Hee Hong, Yoon Jin Cha, Su Jin Heo, Hyung Soon Park, Jee Hung Kim, Jin Gu Lee, Chang Young Lee, Byoung Chul Cho, Hye Ryun Kim

**Affiliations:** ^1^ Division of Medical Oncology, Department of Internal Medicine, Yonsei Cancer Center, Seoul, Korea; ^2^ Graduate School of Medical Science and Engineering, KAIST, Daejeon, Korea; ^3^ Department of Pathology, Yonsei University College of Medicine, Seoul, Korea; ^4^ Department of Thoracic and Cardiovascular Surgery, Yonsei University College of Medicine, Seoul, Korea; ^5^ JE-UK Institute for Cancer Research, JE-UK Co., Ltd., Gumi, Kyungbuk, Korea

**Keywords:** non-small cell lung cancer, epidermal growth factor receptor mutation, first-generation epidermal growth factor receptor-tyrosine kinase inhibitors, liquid biopsy, peptide nucleic acid clamping-assisted fluorescence melting curve analysis

## Abstract

This study was designed to prospectively examine whether peptide nucleic acid clamping-assisted fluorescence melting curve analysis (PANAMutyper™) is feasible for the detection of activating and acquired resistant *epidermal growth factor receptor* (*EGFR*) mutation in plasma. Patients with non-small cell lung cancer harboring activating EGFR mutations who were scheduled to undergo EGFR-tyrosine kinase inhibitors (EGFR-TKIs) were enrolled between September 2011 and March 2015. A total of 102 patients with EGFR-mutated lung cancer were enrolled, 53 had available plasma samples at disease progression, and 28 underwent serial plasma sampling during EGFR-TKI treatment. EGFR-TKI-sensitizing and T790M mutations were detected in the plasma of 68.6% (70/102) at baseline and 30.2% (16/53) at disease progression, respectively. The concordance rates for matched tissue and plasma samples were 80.4% and 90.2% for E19del and L858R mutations at baseline and 56.3% for T790M mutation at disease progression. The sustained presence of plasma *EGFR* mutations four weeks after EGFR-TKI predicted a poor objective response rate (30.0% vs. 87.5%, *P* = 0.025), as well as worse progression-free survival (hazard ratio [HR], 4.381) and overall survival (HR, 5.475). Longitudinal analysis could detect T790M mutations earlier than disease progression based on imaging study (median time from appearance of T790M in plasma samples to progression at imaging scan, 103 days). In conclusion, PANAMutyper™ is reliable for detecting activating and acquired resistant EGFR mutation in plasma, and predicts responses to EGFR-TKI via longitudinal monitoring of EGFR mutation during treatment.

## INTRODUCTION

Activating *epidermal growth factor receptor* (*EGFR*) mutations are predictive biomarkers for response to EGFR-tyrosine kinase inhibitors (EGFR-TKIs; e.g., erlotinib, gefitinib or afatinib) and EGFR-TKIs are the standard first-line therapy for non-small cell lung cancer (NSCLC) with activating *EGFR* mutations [1–3]. Randomized phase III studies have consistently demonstrated that first-line EGFR-TKI therapy improves progression-free survival (PFS) compared with standard cytotoxic chemotherapy in *EGFR* mutated lung cancer patients [2–4]. However, most patients treated with EGFR-TKI ultimately develop disease progression due to acquired resistance via multiple mechanisms [5–7]. Of these mechanisms, *EGFR* T790M mutation accounts for more than 50% of the acquired resistance [8]. Third-generation EGFR-TKIs have shown promising activity against *EGFR* T790M mutation-positive NSCLC and recently osimertinib was approved by the US Food and Drug Administration [9]. Therefore, in the era of third generation EGFR-TKI, the detection of *EGFR* T790M mutation in repeated biopsies at the time of EGFR-TKI failure is indispensable to improving survival outcomes in *EGFR* mutated patients.

Analysis of *EGFR* mutations in tumor tissue is not always possible due to the invasive nature of biopsies, inaccessibility of tumor location, or low quantity and quality of the tissue samples [10, 11]. Moreover, single-site biopsy cannot provide a representative profile of the overall resistance mechanisms for patients with multiple metastatic sites with heterogeneous characteristics [12]. In reality, a monitoring of mutation dynamics during EGFR-TKI through repetitive biopsies is not suitable. Detection of circulating free tumor DNA (ctDNA) in plasma has been considered as a feasible method for diagnosis, prediction of treatment efficacy, and monitoring of recurrence or disease burden in various solid tumors in recent years [13–15]. In meta-analyses, ctDNA has proven to be a highly specific and effective biomarker for the detection of activating *EGFR* mutation in NSCLC [16, 17]. The T790M mutation was also successfully detected by liquid biopsy through analysis of blood samples [18, 19]. Compared to tumor tissue biopsy, liquid biopsy for detecting ctDNA is safer because of its non-invasive nature and more feasible for monitoring tumor dynamics as it is representative of multiple tumor sites [20].

Peptide nucleic acids (PNA) are synthetic polymers that bind tightly to a complementary sequence in DNA [7, 21]. Despite its lack of ability to detect new mutations, PNA-mediated polymerase chain reaction (PCR) clamping assay has advantages of sensitivity, simplicity, and speed for detecting previously known mutations and thus, real-time PCR with PNA has been widely used to detect *EGFR* mutation. Recently, the Korean Food and Drug Administration approved the PNA Clamp^™^ *EGFR* Mutation Detection kit (PANAGENE Inc., Daejeon, Korea) as a standard screening method for *EGFR* mutation in lung cancer patients [21, 22]. To increase the sensitivity of the PNA clamp method in order to detect even in plasma ctDNA, PNA clamping-assisted fluorescence melting curve analysis (PANAMutyper^™^ *EGFR* kit) was newly developed using a fluorescence melting curve in addition to PNA clamping.

In this study, we prospectively evaluated whether PNA clamping-assisted fluorescence melting curve analysis (PANAMutyper^™^) can accurately detect activating and acquired resistance *EGFR* mutations in plasma ctDNA derived from NSCLC patients. Additionally, we aimed to explore dynamic changes in *EGFR* mutation profiles and the appearance of acquired resistance during EGFR-TKI in *EGFR* mutated lung cancer patients.

## RESULTS

### Patient characteristics and treatment outcomes for EGFR-TKI

**Figure 1:**
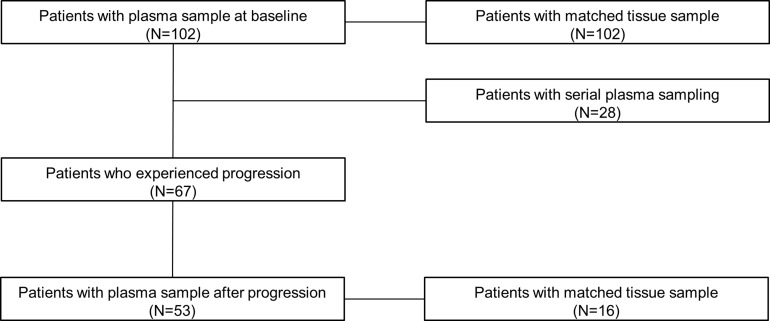
Patients with available tissue or plasma samples before administration of EGFR-TKI, during course of treatment, and after progression

A total of 102 patients with NSCLC harboring activating *EGFR* mutations were enrolled in this prospective trial of first-generation EGFR-TKI between September 2011 and March 2015 at Yonsei Cancer Center in Korea (Figure 1). Baseline examination of *EGFR* mutations was performed in 102 patients using matched tumor tissues and plasma samples (Table 1). The majority were female (62/102, 60.8%), never smokers (71/102, 69.6%) with extra-thoracic metastatic disease (M1b) (71/102, 69.6%) who received gefitinib (81/102, 79.4%) as the first-line treatment (72/102, 70.6%). The most common mutation identified was E19del (57/102, 55.9%) and T790M was not detected in any patients at baseline. Objective responses were observed in 64 patients (62.8%), including a complete response in one patient (1/102, 1.0%) and a partial response in over half of the patients (63/102, 61.8%). The median PFS was 13.6 months (95% confidence interval [CI], 11.6–15.6 months) and the median overall survival (OS) was 28.6 months (95% CI, 12.2–45.0 months). During a median follow-up period of 36.4 months (95% CI, 31.8–41.1 months), disease progression and death events occurred in 67 (65.7%) and 40 (39.2%) patients, respectively. Dynamic changes in *EGFR* mutations were analyzed in 28 patients using serial sampling (Supplementary Table 1). At the time of disease progression, 53 blood samples were available, including 16 paired tissue biopsies and blood samples. All causes of death were related to disease progression.

**Table 1:** Baseline characteristics and treatment outcomes of patients (*N* = 102)

Variables	*N*	%
Age (years)		
Median	61	
Range	33–84	
Gender		
Male	40	39.2
Female	62	60.8
Smoking history		
Never-smoker	71	69.6
Ever smoker	31	30.4
Stage		
M0/M1a	31	30.4
M1b	71	69.6
Type of *EGFR* mutation		
E19del	57	55.9
L858R	45	44.1
Line of treatment		
1st	72	70.6
2nd	30	29.4
TKI		
Erlotinib	21	20.6
Gefitinib	81	79.4
Best response		
Complete response	1	1.0
Partial response	63	61.8
Stable disease	33	32.4
Progressive disease	2	2.0
Not assessable	3	2.9

Abbreviations: E19del, exon 19 deletion; *EGFR*, epidermal growth factor receptor; TKI, tyrosine kinase inhibitor.

### Performance of the platform

*EGFR* E19del or L858R mutations were detected in baseline plasma samples prior to treatment in 68.6% (70/102) of the patients (Table 2). Sensitivity of M1b disease was higher than that of M0/M1a disease (83.1% vs. 35.5%, *P* < 0.001). Likewise, median copy numbers of activating *EGFR* mutation were higher in M1b than in M0/M1a disease (1422.7 copies/mL vs. 431.5 copies/mL, *P* < 0.001). Concordance rate, sensitivity, and negative predictive value (NPV) between plasma ctDNA and matched tissue were 80.4% (80/102), 61.8% (35/57), and 67.2% (45/67) for E19del and 90.2% (92/102), 77.8% (35/45), and 85.1% (57/67) for L858R. E19del mutations were not detected in plasma from patients with L858R mutations and *vice versa*, yielding a specificity and positive predictive value (PPV) of 100% for both subtypes of *EGFR* mutation.

**Table 2:** Comparison between plasma and tissue samples at baseline

		Tissue EGFR mutation		
		E19del	L858R	Total patients
ctDNA *EGFR* mutation	E19del	35	0	35
	L858R	0	35	35
	Wild	22	10	32
	Total patients	57	45	102

Abbreviations: ctDNA, circulating free tumor DNA; E19del, exon 19 deletion; *EGFR*, epidermal growth factor receptor.

### Monitoring of acquired resistance during serial sampling and at disease progression

**Figure 2:**
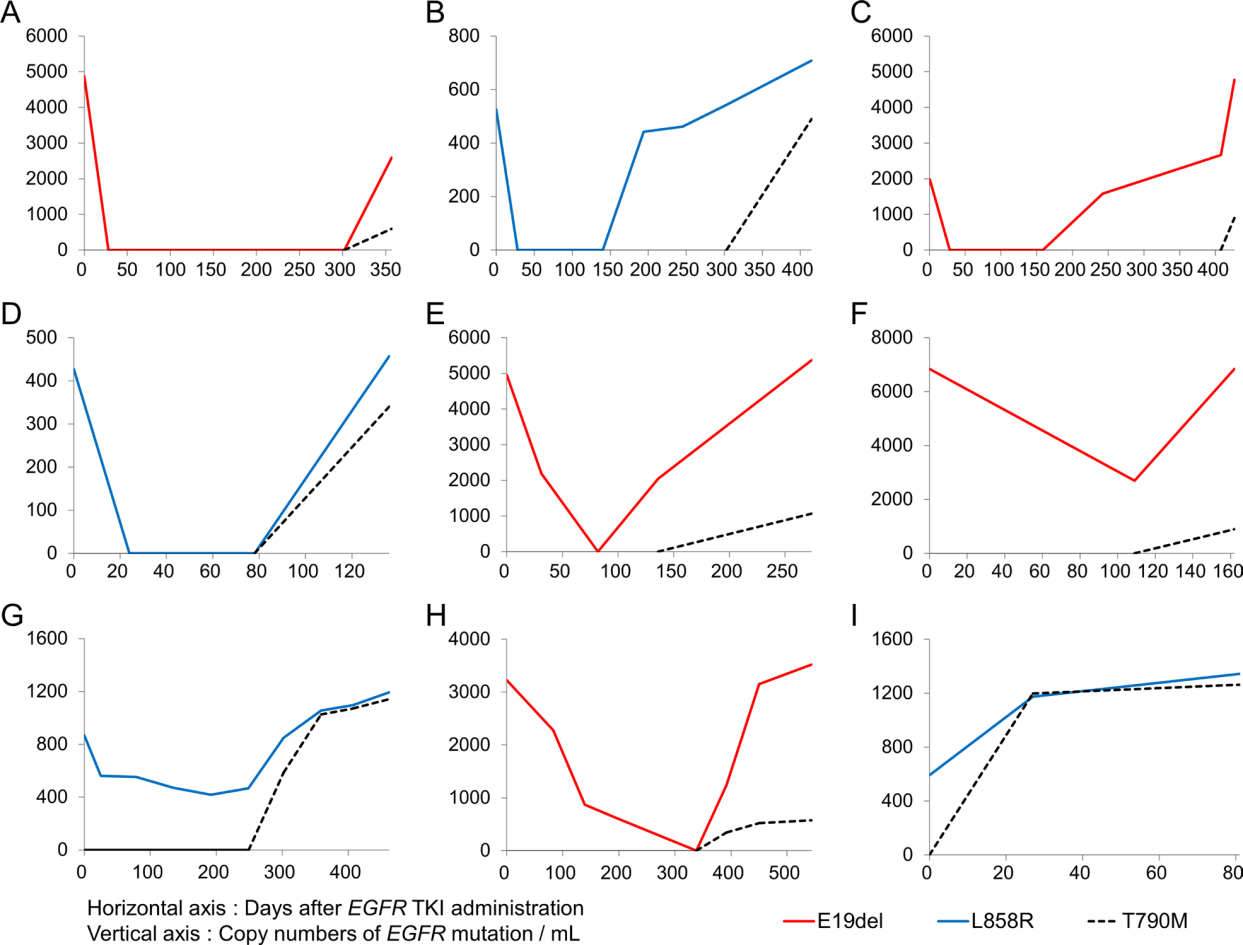
Representatives for longitudinal monitoring of activating and resistant EGFR mutations in ctDNA Each graph (**A**–**E**) represents each patient.

A decrease in copy numbers of activating plasma *EGFR* mutation (E19del or L858R) was seen in 94.4% of the patients (17/18) four weeks after administration of EGFR-TKI, except one patient who progressed within 81 days and revealed detectable plasma T790M at 28 days after treatment (Figure 2). Genomic profiling of tumor tissue from this non-responder by next-generation sequencing revealed mutations in TP53, as well as STK11, which is known to be associated with *de novo* resistance to EGFR-TKI [23]. Negative conversion of detectable plasma *EGFR* mutation was seen in 44.4% of the patients (8/18), and the median rates of decrease in copy numbers was 67.9% (range, 11.3%–100.0%). Re-emergence or increasing copy numbers of activating *EGFR* mutation in plasma was also observed before objective disease progression based on imaging scans according to Response Evaluation Criteria in Solid Tumors (RECIST) 1.1 guidelines. Emergence of acquired resistance (T790M) was always associated with disease progression which was later evaluated based on imaging scan. Median time from appearance of T790M in plasma samples to progression at imaging scan was 103 days (range, 0–169 days).

Among the 67 patients who experienced disease progression after EGFR-TKI treatment, 53 patients had blood plasma samples available at the time of disease progression (Table 3). The prevalence of T790M mutation in plasma was 30.2% (16/53). Comparison between matched tumor tissues by repeated biopsy and plasma at disease progression was performed in 16 patients (Table 4). The prevalence of the T790M mutation was 37.5% (6/16) for tissue samples and 31.3% (5/16) for plasma samples. The concordance rate of T790M mutations between tissue and plasma samples was 56.3% (9/16). Seven cases with discrepancies in T790M mutations included three patients who were positive for the T790M mutation in plasma but negative in tissue samples and four patients who showed the reverse. In examination of both tissue and plasma samples, resistance mechanisms to EGFR-TKIs were driven by secondary T790M mutations (9 patients), *c-MET* amplification by fluorescent *in situ* hybridization (1 patient), small cell transformation (1 patient), epithelial-to-mesenchymal transition by histologic analysis (1 patient), and unknown causes (4 patients).

**Table 3:** Subtypes of ctDNA EGFR mutation at disease progression (*N* = 53)

ctDNA *EGFR* mutation	***N***	**%**
E19del	8	15.1
E19del, T790M	9	17.0
L858R	9	17.0
L858R, T790M	6	11.3
T790M	1	1.9
Wild	20	37.7

Abbreviations: ctDNA, circulating free tumor DNA; E19del, exon 19 deletion; *EGFR*, epidermal growth factor receptor.

**Table 4:** Comparison between plasma and tissue samples at disease progression

E19del		Tissue *EGFR* mutation					
		E19del, T790M	L858R	L858R, T790M	T790M	Total patients	
ctDNA *EGFR* mutation	E19del	1	1	0	0	0	2
	E19del,T790M	2	1	0	0	0	3
	L858R	0	0	1	2	0	3
	L858R,T790M	0	0	1	1	0	2
	Wild	5	0	0	0	1	6
	Total patients	8	2	2	3	1	16

Abbreviations: ctDNA, circulating free tumor DNA; E19del, exon 19 deletion; *EGFR*, epidermal growth factor receptor.

**Figure 3:**
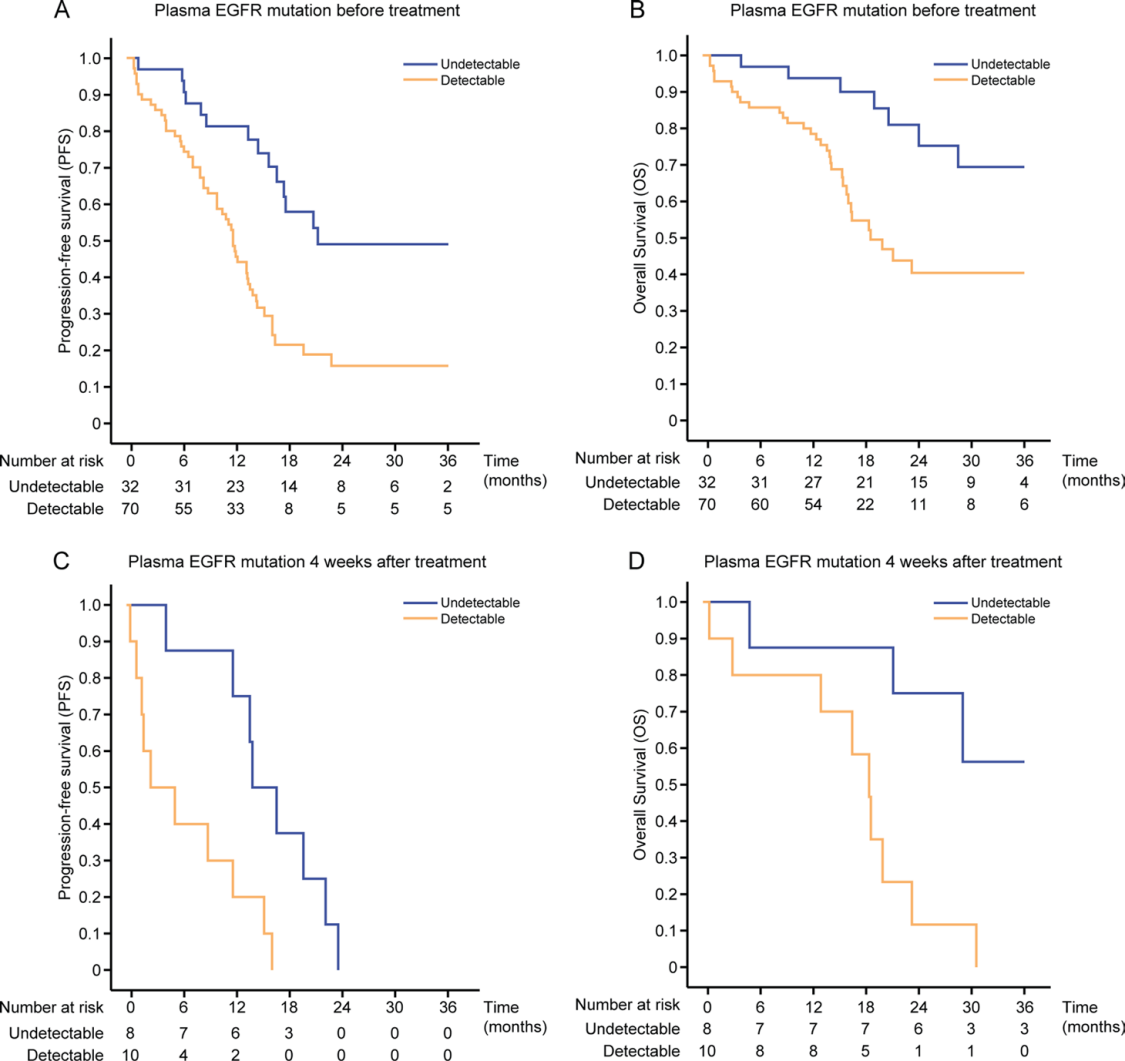
Kaplan–Meier survival estimates for patients according to plasma *EGFR* mutation PFS (**A**) and OS (**B**) by ctDNA status at baseline. PFS (**C**) and OS (**D**) by ctDNA status four weeks after treatment.

Prognostic and predictive value of ctDNA from blood plasma samples. Median PFS was 11.9 months for patients with detectable plasma *EGFR* mutations before treatment *versus* 21.4 months for those without detectable mutations (hazard ratio [HR], 2.964; 95% CI, 1.624–5.409; *P* < 0.001; Figure 3A) and median OS was 18.8 *versus* 37.6 months, respectively (HR, 2.790; 95% CI, 1.274–6.110; *P =* 0.007; Figure 3B). Objective response rates were not significantly different between the two groups. In analysis adjusted for other demographic and clinicopathologic variables, detectable plasma *EGFR* mutation before EGFR-TKI was independently associated with worse PFS (HR, 2.694; 95% CI, 1.416–5.125) and OS (HR, 2.436; 95% CI, 1.040–5.704), respectively (Table 5). Of the 28 patients who underwent serial plasma sampling, 18 patients (64.3%) had detectable *EGFR* mutations before EGFR-TKI treatment. Moreover, the presence of *EGFR* mutation after treatment might predict poor prognosis of these patients. Median PFS was 2.7 months for patients with detectable plasma *EGFR* mutation, even after four weeks of treatment *versus* 14.1 months for patients without detectable mutations (HR, 4.381; 95% CI, 1.340–14.316; *P* = 0.016; Figure 3C), whereas median OS was 18.6 months *versus* not reached, respectively (HR, 5.475; 95% CI, 1.425–21.035; *P* = 0.020; Figure 3D). Objective response rates after four weeks of treatment were 30.0% for patients with detectable plasma *EGFR* mutation and 87.5% for patients without detectable mutation *P* = 0.025).

**Table 5:** Multivariate analysis of prognostic factors with progression-free survival and overall survival

		Progression-free survival		Overall survival	
		HR (95% CI)	*P* value	HR (95% CI)	*P* value
Gender			0.239		0.237
	Female	Ref		Ref	
	Male	0.580 (0.234–1.435)		0.444 (0.116–1.706)	
Smoking history			0.123		0.271
	Never-smoker	Ref		Ref	
	Ever smoker	2.139 (0.813–5.629)		2.230 (0.534–9.312)	
Stage			0.248		0.410
	M0/M1a	Ref		Ref	
	M1b	1.502 (0.753–2.994)		1.466 (0.590–3.643)	
Type of *EGFR* mutation			0.983		0.100
	E19del	Ref		Ref	
	L858R	0.995 (0.598–1.654)		1.981 (0.877–4.476)	
Line of treatment			0.302		0.099
	1st	Ref		Ref	
	2nd	1.321 (0.778–2.243)		1.798 (0.896–3.606)	
TKI			0.803		0.736
	Gefitinib	Ref		Ref	
	Erlotinib	1.087 (0.566–2.086)		1.149 (0.512–2.581)	
Presence of pretreatment ctDNA			0.003		0.040
	No	Ref		Ref	
	Yes	2.694 (1.416–5.125)		2.436 (1.040–5.704)	

Abbreviations: CI, confidence interval; ctDNA, circulating free tumor DNA; E19del, exon 19 deletion; HR, hazard ratio; Ref, referent; TKI, tyrosine kinase inhibitor.

**Figure 4:**
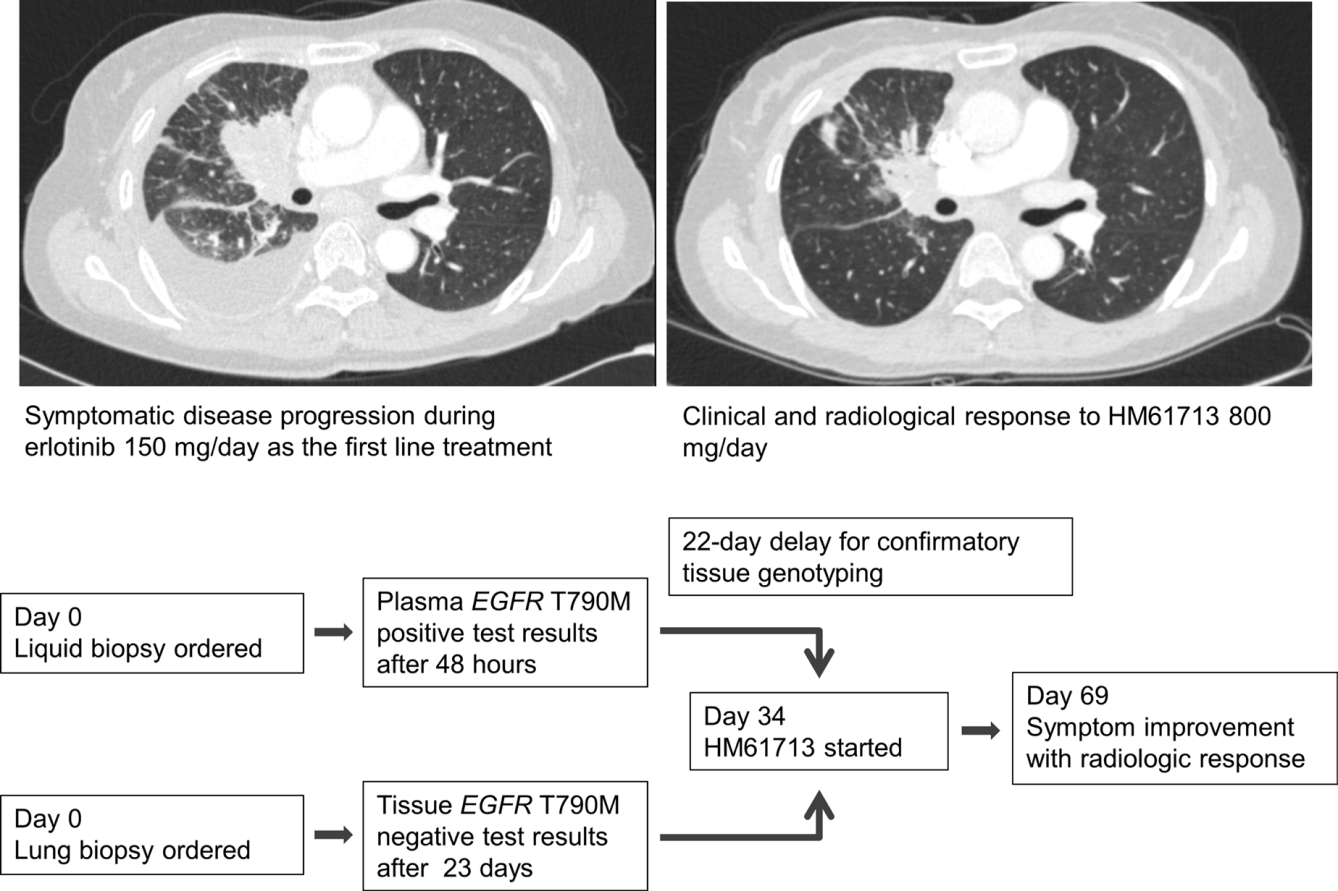
A patient with metastatic NSCLC with acquired resistance to erlotinib who showed a partial response to third-generation TKI

In the case of a 56-year-old female who developed acquired resistance after 9 months’ treatment with erlotinib (Figure 4), a plasma sample obtained at disease progression revealed both EGFR E19del and T790M mutations within 48 hours after sampling. A confirmatory lung biopsy was also performed, which revealed only an EGFR E19del mutation 23 days after the procedure. Treatment with a third-generation TKI, HM61713, was subsequently initiated and the patient exhibited a partial response to therapy, which continues to be maintained. The benefits of liquid biopsy including delicate reflection of tumor heterogeneity as well as fast turn-around time can be seen in this case.

## DISCUSSION

This is the first prospective study demonstrating the feasibility of PNA clamping-assisted fluorescence melting curve analysis (PANAMutyper^™^) of plasma ctDNA derived from NSCLC patients with activating and acquired resistant *EGFR* mutations. The overall sensitivity and specificity of this platform were 68.6% and 100% at baseline, respectively. Longitudinal plasma analysis was found to detect acquired resistance earlier than that of tumor tissue biopsies, and demonstrated that the prevalence of activating and resistant mutations increased over time before progression. Presence of *EGFR* mutations in plasma prior to EGFR-TKI treatment was associated with impaired survival outcomes. In most patients, DNA copy numbers of activating EGFR mutation decreased in plasma within four weeks after EGFR-TKI. Interestingly, the presence of detectable plasma *EGFR* mutation four weeks after EGFR-TKI was associated with lower objective response rates and shorter PFS and OS than the absence of mutation.

Recent studies suggest that *EGFR* mutation analysis with ctDNA through liquid biopsy can be an alternative detection method for patients who cannot receive invasive procedures for tissue analysis [19, 24]. High specificity was observed consistently in several different platforms, although false-negative results are still an important issue regarding the diagnostic value of blood-based analysis in clinical practice. Kim *et al.* reported that the overall sensitivity of *EGFR* mutations in ctDNA was 17.1% with PNA-mediated PCR clamping [25]. Similarly, the sensitivity of PNA PCR clamping was low at 22.2% (4/18) in our independent analysis (Supplementary Table 2). On the other hand, by incorporating melting curve analysis to PNA-mediated PCR clamping method, the sensitivity of this platform (PANAMutyper^™^) increased up to nearly 70%, similar to previous studies conducted by Han *et al* [26]. This result was comparable to previous studies using the non-digital amplification refractory mutation system (ARMS) assay [27], allele-specific PCR assay [28], next generation sequencing (NGS) [29], and droplet digital PCR (ddPCR) assay [30]. In comparison with other methods, PANAMutyper^™^ can be performed simply with real-time PCR and finished in a brief time [7]. Furthermore, it can simultaneously detect multiple mutations while maintaining high sensitivity and specificity [31].

Although EGFR-TKI prolonged survival outcome of patients with activating *EGFR* mutation, this benefit was not distributed equally among patients [1, 3]. Hence, plasma-based *EGFR* mutation analysis has been used to monitor responses to EGFR-TKI in previous studies. Mok *et al.* reported an association of negative conversion of *EGFR* mutation of ctDNA 12 weeks after treatment along with prolonged PFS and OS [28]. Lee *et al.* also showed that detectable *EGFR* mutation in plasma during the course of treatment was associated with impaired outcomes [30]. In line with these previous studies, the predictive value of plasma *EGFR* mutation after treatment was proven in the present study. In addition to confirming the correlation of PFS and OS with dynamic changes in *EGFR* mutation status, we also demonstrated that radiologic response according to RECIST criteria was correlated with post-treatment plasma *EGFR* mutation status. We evaluated the predictive value of an early response reflected by ctDNA at four weeks after treatment, which is earlier than other previous studies [28, 32]. Considering that outcomes for patients who continued to have detectable plasma *EGFR* mutation four weeks after treatment were relatively poor, assessment of plasma *EGFR* mutation status four weeks after treatment can help in timely discrimination of patients who may benefit from continuing EGFR-TKI.

T790M is the most common mutation associated with acquired resistance to first-generation EGFR-TKIs [8]. The development of third-generation TKIs has substantially benefitted patients with acquired resistance to erlotinib or gefitinib [9, 19]. Because of the limitation of repeated biopsies, several studies have evaluated acquired resistance using non-invasive blood-based analyses [15, 24]. In the present study, the T790M mutation was found in 16 of 53 patients (30.2%), and was detectable as early as six months before objective disease progression. The relative proportion of T790M mutations increased after the first appearance of such acquired resistance, and all patients with detectable T790M in serial plasma samples eventually experienced disease progression.

When we compared *EGFR* mutation results of tumor tissues and plasma samples at disease progression, 18.8% of patients (3/16) had detectable T790M in plasma that was not detected via tissue biopsy. This discrepancy could result from the heterogeneous nature of metastatic tumor deposits [33, 34]. The concordance rate between plasma and tissue for E19del or L858R before EGFR-TKI was higher than that for T790M at disease progression (80.4% and 90.2%, respectively, vs. 56.3%). Therefore, confounding results of repeat biopsy can be supplemented by non-invasive blood-based methods. Studies focusing on the clinical relevance of early detection of resistant mutations in ctDNA or discrepancy of T790M between tumor tissue and plasma samples are underway using ARMS and ddPCR methods (NCT02418234).

Unfortunately, the predictive role of T790M in plasma could not be fully assessed in this study, because of the small number of patients who underwent plasma sampling at disease progression or received subsequent treatment with the third-generation TKI. In addition, repeated tumor biopsy after disease progression was performed in only a small number of patients (16/67, 23.9%), mainly because of clinical deterioration.

In conclusion, PNA clamping-assisted fluorescence melting curve analysis (PANAMutyper^™^) in plasma samples is a feasible and effective method for diagnosis of activating mutation, prediction of treatment response, and monitoring of acquired resistance during EGFR-TKI treatment. Larger studies on the clinical relevance of T790M genotyping based on this platform are warranted to confirm our results. Ultimately, prospective clinical trials exploring the correlation with response to third-generation EGFR-TKI therapy could help validate the usefulness of this platform for T790M genotyping in clinical practice.

## MATERIALS AND METHODS

### Study design

This study was an exploratory trial to evaluate the performance of a newly developed platform (PANAMutyper^™^) for detecting activating and acquired resistant *EGFR* mutation in plasma from NSCLC patients harboring *EGFR* mutation during EGFR-TKI. Patients with NSCLC harboring activating *EGFR* mutations were enrolled in a prospective trial of first-generation EGFR-TKI (gefitinib or erlotinib) at Yonsei Cancer Center in Korea. Activating *EGFR* mutations were defined as mutations known to be associated with EGFR-TKI sensitivity, including E19del and L858R. Patients with available archival tissue and those with measurable lesions at baseline were enrolled. Baseline tissue and blood plasma samples were collected before EGFR-TKI administration. Serial plasma sampling and tissue or plasma sampling at the time of progression were optional in our trial. This study was approved by the Institutional Review Board and the ethics committee of Yonsei Cancer Center. All patients provided written informed consent for study participation and genetic analysis.

### Study objectives

The primary objective was to assess the diagnostic utility of plasma sampling using matched tissue and plasma samples. Sensitivity, specificity, PPV, NPV, and concordance rate were analyzed in comparison to specific tissue *EGFR* mutation status. Secondary objectives included assessment of the prognostic and predictive value of plasma *EGFR* mutations at baseline and serial sampling, monitoring acquired resistance, and comparison between tissue and plasma samples upon disease progression.

### Treatment and evaluation of response

Patients received erlotinib at 150 mg/d or gefitinib at 250 mg/d. Evaluation of response by computed tomography scans was performed four weeks after the baseline study, and then every eight weeks thereafter according to the RECIST 1.1 guidelines. Serial sampling was performed at the time of response evaluation. Treatment was continued until disease progression, intolerable toxicity, or withdrawal of consent.

### Data collection

Medical records and radiologic images of all patients were collected to evaluate demographic and clinicopathologic parameters, tumor response, PFS, and OS. Never smokers were defined as those with a lifetime smoking dose of less than 100 cigarettes. PFS was measured from the first day of treatment with EGFR-TKI to tumor progression or death. OS was measured from the first date of treatment with EGFR-TKI until the date of death. Patients were censored at the last visiting if alive and progression-free. Sensitivity, specificity, PPV, NPV, and concordance rate were calculated for E19del, L858R, and T790M mutations as described in Supplementary Table 3.

### DNA extraction

Tumor DNA was extracted using the Maxwell R 16 FFPE purification kit (Promega, Mannheim, Germany) and ctDNA was extracted from 1 mL of plasma using the QIAamp MinElute Virus Spin kit (QIAGEN, Hilden, Germany) according to the manufacturers’ protocols.

### *EGFR* mutation analysis for tissue

We used the PNAClamp^™^ *EGFR* Mutation Detection kit (PANAGENE Inc., Daejeon, Korea) to detect *EGFR* mutations by real-time PCR. All reactions were performed in 20 µl volumes containing template DNA, primer and PNA probe sets, and fluorescence PCR master mix. All reagents were included in the kit. Real-time PCR reactions of PNA-mediated clamping PCR were performed using a CFX 96 system (Bio-Rad, Hercules, CA, USA). PCR cycling conditions were a 5 min hold at 94°C, followed by 40 cycles of 94°C for 30 sec, 70°C for 20 sec, 63°C for 30 sec, and 72°C for 30 sec. The lowest mutation allele frequency for the E19del, L858R, and T790M mutations measured by the PNAClamp^™^ *EGFR* was set as 1.0%, corresponding to one mutated copy in 100 copies of wild type DNA (Supplementary Figure 1).

### *EGFR* mutation analysis for plasma

The PANAMutyper^™^ *EGFR* kit was used for mutation detection. The PANAMutyper^™^ *EGFR* kit is a newly developed mutation detection kit designed to detect 47 different *EGFR* variants in exons 18–21 with high sensitivity using PNA clamping-assisted fluorescence melting curve analysis for mutation detection and genotyping. All reactions were performed in a total volume of 25 µl that contained 10–25 ng of DNA templates, primer and PNA probe sets, and PCR master mix. All reagents used were included with the kit. PCR was performed under the following conditions: 50°C for 2 min and 95°C for 15 min as two holding periods; 15 cycles of 95°C for 30 sec, 70°C for 20 sec, 63°C for 60 sec; 35 cycles of 95°C for 10 sec, 53°C for 20 sec, 73°C for 20 sec; and a melting curve step (from 35°C to 75°C with gradual increment for 0.5°C for 3 sec). Fluorescence was measured on all four channels (FAM, ROX, Cy5, and HEX). Melting peaks were derived from the melting curve data. Mutations were detected by the melting temperature of each tube for each fluorescent dye as shown in Supplementary Table 4.

The total amount of plasma volume required for assay was 1 mL and all samples were successfully genotyped. The median amounts of amplifiable DNA extracted from plasma for E19del, L858R, and T790M were 711.6 (range, 0–8707.3), 206.1 (range, 0–1721.7), and 68.5 (range, 0–1262.9) copies/mL, respectively. The lowest mutation allele frequency for the E19del, L858R, and T790M mutations measured by the PANAMutyper^™^ was set as 0.01%, corresponding to one mutated copy in 10,000 copies of wild type DNA (Supplementary Figure 2). This cut-off value yielded 100% specificity when plasma derived from 10 healthy volunteers (ZenBio Inc., Durham, NC, USA) and normal genomic DNA (Horizon Dx Inc., Cambridge, UK) was tested (Supplementary Figure 3). All results were available within three hours with this ready-to-use kit.

### Statistical analysis

Fisher’s exact test and Wilcoxon rank-sum test were used for categorical and continuous variables. Survival distribution was estimated by the Kaplan–Meier method, and the Cox proportional hazards model was used to analyze the effect of specified risk factors on survival. Results were considered statistically significant at a *P* value < 0.05. All analyses were performed using statistical software package SPSS for Windows software, version 22.0 (SPSS Inc., Chicago, IL, USA).

## SUPPLEMENTARY MATERIALS FIGURES AND TABLES


