# Supplementary material for: Detection of activating and acquired resistant mutation in plasma from EGFR-mutated NSCLC patients by peptide nucleic acid (PNA) clamping-assisted fluorescence melting curve analysis
Source: Oncotarget. 2017 May 10;8(39):65111–22. doi: 10.18632/oncotarget.17786 (PMC5630316; doi:10.18632/oncotarget.17786)
Supplement: Supplementary file 1 [file oncotarget-08-65111-s001.pdf]

Detection of activating and acquired resistant mutation in plasma from EGFR-mutated NSCLC patients by peptide nucleic acid (PNA) clamping-assisted fluorescence melting curve analysis

SUPPLEMENTARY MATERIALS

A

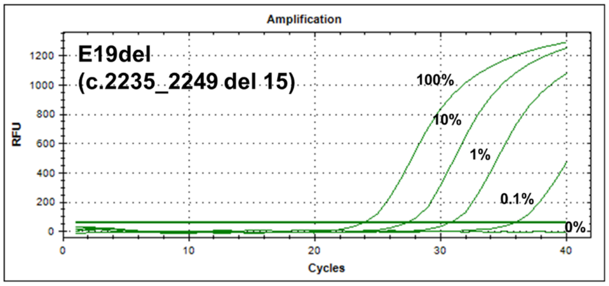

Frequency	100%	10%	1%	0.1%	0%
Cycle (Ct)	23.88	27.27	30.73	35.92	38
△Ct	10.12	6.73	3.27	-1.92	-4

B

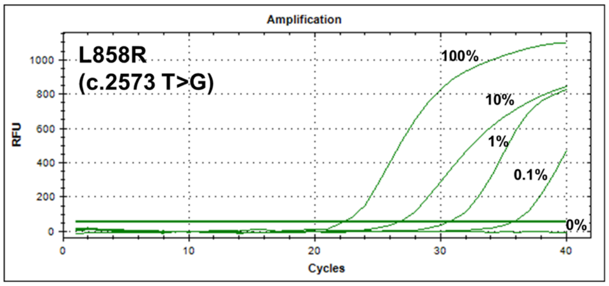

Frequency	100%	10%	1%	0.1%	0%
Cycle (Ct)	22.37	26.81	30.75	35.92	38
△Ct	10.63	6.19	2.25	-2.92	-5

C

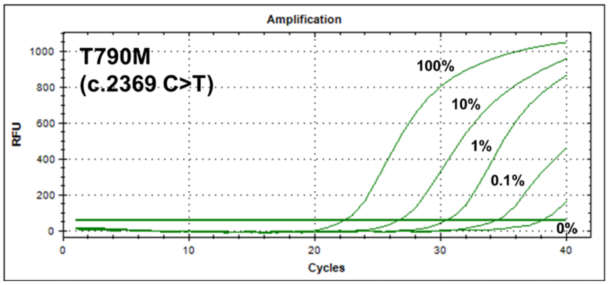

Frequency	100%	10%	1%	0.1%	0%
Cycle (Ct)	22.33	26.68	30.47	34.39	38.08
△Ct	10.67	6.32	2.53	-1.39	-5.08

Supplementary Figure 1: Detection thresholds of PNAClamp™ *EGFR* Mutation Detection kit for E19del (**A**), L858R (**B**), and T790M (**C**) mutations.

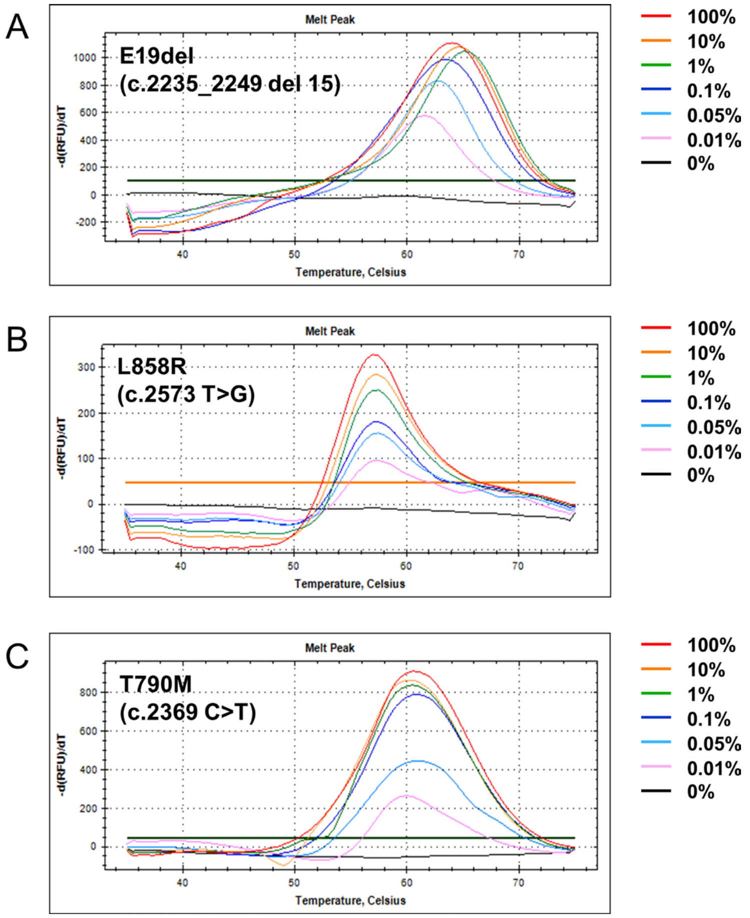

Supplementary Figure 2: Detection thresholds of PANAMutyper™ *EGFR* kit for E19del (**A**), L858R (**B**), and T790M (**C**) mutations.

A

Normal DNA	Pealk	E19del mutant DNA	Peak
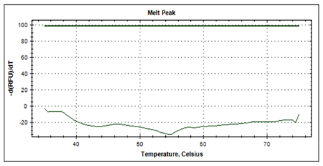	Not available	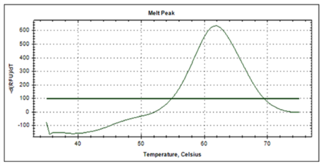	62 ℃
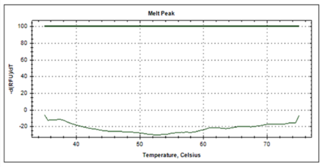	Not available	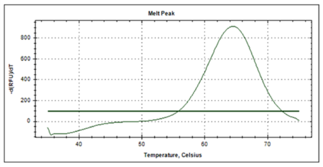	64.5 ℃
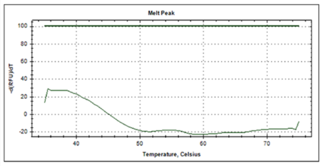	Not available	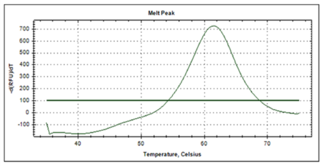	61.5 ℃

B

Normal DNA	Peak	L858R mutant DNA	Peak
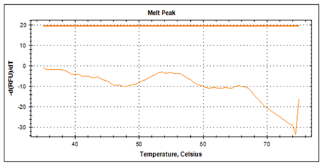	Not available	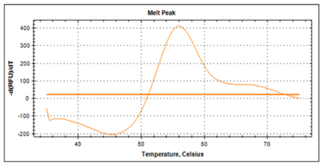	56 ℃
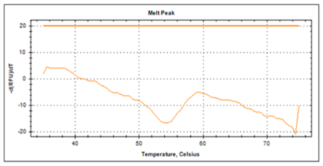	Not available	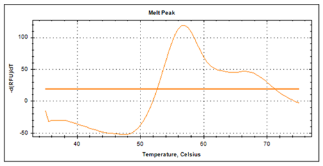	57 ℃
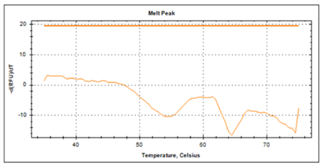	Not available	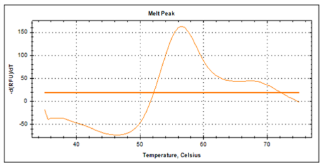	56.5 ℃

C

Normal DNA	Peak	T790M mutant DNA	Peak
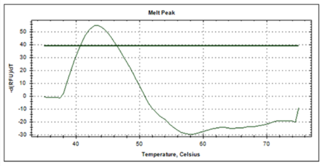	Not available	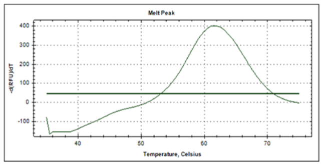	61.5 ℃
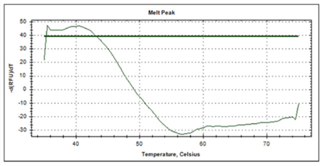	Not available	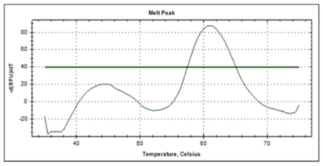	61 ℃
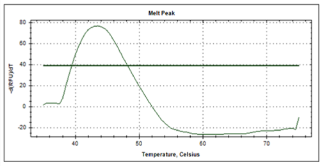	Not available	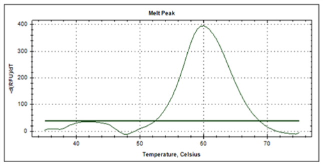	60 ℃

Supplementary Figure 3: Melting curve analysis of E19del (**A**), L858R (**B**), and T790M (**C**) using normal genomic DNA and mutant DNA using PANAMutyper™ *EGFR* kit.

Supplementary Table 1: Characteristics of patients who underwent serial plasma sampling (*N* = 28)

Patients number	ctDNA *EGFR* mutation			Time to emergence of plasma T790M mutation (days)	Time to disease progression according to RECIST 1.1 (days)
	Baseline	4 weeks after treatment	Progression		
1	L858R	Wild	L858R	-	133
2	Wild	Wild	L858R	-	131
4	E19del	E19del	E19del	-	274
7	Wild	Wild	E19del	-	267
12	E19del	Wild	E19del	-	594
17	L858R	L858R, T790M	L858R,T790M	28	81
19	L858R	L858R	L858R	-	50
24	Wild	Wild	Wild	-	711
27	E19del	Wild	T790M	544	713
32	E19del	Wild	Wild	-	668
33	L858R	L858R	L858R	-	358
35	Wild	Wild	Wild	-	199
37	L858R	L858R	L858R	-	11
39	Wild	Wild	Wild	-	402
43	E19del	E19del	E19del,T790M	44	56
44	E19del	E19del	E19del,T790M	162	162
53	Wild	Wild	Wild	-	412
54	E19del	E19del	E19del,T790M	337	489
59	E19del	Wild	E19del,T790M	422	422
62	Wild	Wild	Wild	-	282
64	L858R	L858R	L858R,T790M	301	461
66	Wild	Wild	Wild	-	193
78	E19del	Wild	Wild	-	505
80	E19del	Wild	E19del,T790M	204	356
82	Wild	Wild	Wild	-	827
94	Wild	Wild	Wild	-	551
97	L858R	L858R	L858R	-	33
99	L858R	Wild	L858R	-	414

Abbreviations: ctDNA, circulating free tumor DNA; E19del, exon 19 deletion; *EGFR*, epidermal growth factor receptor; RECIST, Response Evaluation Criteria in Solid Tumors.

Supplementary Table 2: Comparison between PNA clamping and PNA clamping-assisted fluorescence melting curve analysis ($N = 18$)

Patients number	Tissue *EGFR* mutation	ctDNA *EGFR* mutation	
		PNA clamping only	PNA clamping-assisted fluorescence melting curve analysis
4	E19del	E19del	E19del
10	L858R	Wild	L858R
17	L858R	Wild	L858R
22	L858R	Wild	L858R
26	E19del	E19del	E19del
30	E19del	Wild	E19del
39	E19del	Wild	Wild
47	E19del	Wild	E19del
54	E19del	Wild	E19del
55	E19del	Wild	Wild
60	E19del	E19del	E19del
61	L858R	L858R	L858R
62	E19del	Wild	Wild
63	E19del	Wild	Wild
79	E19del	Wild	E19del
81	E19del	Wild	Wild
83	L858R	Wild	Wild
92	L858R	Wild	L858R
Sensitivity	-	22.2% (4/18)	66.7% (12/18)

Abbreviations: ctDNA, circulating free tumor DNA; E19del, exon 19 deletion; *EGFR*, epidermal growth factor receptor; PNA, peptide nucleic acid.

Supplementary Table 3: Calculation of sensitivity, specificity, PPV, NPV, and concordance rate for three subtypes of *EGFR* mutation (E19del, L858R, and T790M)

		Subtypes of *EGFR* mutation in tissue	
		**Positive**	**Negative**
**Subtypes of *EGFR* mutation in ctDNA**	**Positive**	**a**	**b**
	**Negative**	**c**	**d**

Sensitivity	a / (a + c)
Specificity	d / (b + d)
PPV	a / (a + b)
NPV	d / (c + d)
Concordance rate	(a + d) / (a + b + c + d)

Abbreviations: ctDNA, circulating free tumor DNA; E19del, exon 19 deletion; *EGFR*, epidermal growth factor receptor; NPV, negative predictive value; PPV, positive predictive value.

Supplementary Table 4: Criteria of mutation detection according to the fluorescent dye and melting temperature

Reagent	Fluorescent dye	Melting temperature	Assessment	
			Amino acid change	Nucleotide change
T790M	HEX	58°C~ 62°C	p.T790M	c.2369 C>T
L858R	ROX	55.5°C~ 59.5°C	p.L858R	c.2573 T>G
		44°C ~ 47°C	p.L858R	c.2573_2574 TG>GT
E19del	HEX	58°C~ 67°C	Deletions (29)	Different 29 mutations
S768I	HEX	58°C~62°C	p.S768I	c.2303 G>T
G719X	FAM	57°C~60°C	p.G719A	c.2156 G>C
		46°C~48.5°C	p.G719S	c.2155 G>A
		50°C~53°C	p.G719C	c.2155 G>T
L861Q	ROX	48.5°C~53.5°C	p.L861Q	c.2582 T>A
EIC	Cy5	59°C~ 63°C	Internal control	

Abbreviations: E19del, exon 19 deletion; EIC, epidermal growth factor receptor internal control; FAM 5′ 6-Carboxyfluorescein; HEX, 6-Hexachlorofluorescein; ROX, 5(6)-Carboxy-X-Rhodamine.
